# Outcomes of Primary Furlow Double-Opposing Z-plasty for the Treatment of Symptomatic Submucous Cleft Palate

**DOI:** 10.1097/SCS.0000000000009385

**Published:** 2023-05-24

**Authors:** Veera Pitkänen, Anika Szwedyc, Suvi Alaluusua, Ahmed Geneid, Pia Vuola, Anne Saarikko

**Affiliations:** * Department of Plastic Surgery, Cleft and Craniofacial Center; † Department of Otolaryngology and Phoniatrics - Head and Neck Surgery, Helsinki University Hospital and University of Helsinki, Finland

**Keywords:** Double-opposing Z-plasty, Furlow, palatoplasty, submucous cleft palate, velopharyngeal insufficiency, VPI

## Abstract

**Background:**

Submucous cleft palate (SMCP) requires surgical repair if symptomatic. The Furlow double-opposing Z-plasty is the preferred method in Helsinki cleft center.

**Aims:**

To assess the efficacy and complications of Furlow Z-plasty in the treatment of symptomatic SMCP.

**Methods:**

This retrospective study reviewed documentation of 40 consecutive patients with symptomatic SMCP who underwent primary Furlow Z-plasty by 2 high-volume cleft surgeons at a single center between 2008 and 2017. Patients underwent perceptual and instrumental evaluation of velopharyngeal function (VPF) by speech pathologists preoperatively and postoperatively.

**Results:**

The median age at Furlow Z-plasty was 4.8 years (SD 2.6, range 3.1–13.6). The overall success rate, including postoperative competent or borderline competent VPF, was 83%, and 10% required secondary surgery for residual velopharyngeal insufficiency. The success rate was 85% in nonsyndromic, and 67% in syndromic patients with no significant difference (*P*=0.279). Complications arose in only 2 (5%) patients. No children were found to have obstructive sleep apnea postoperatively.

**Conclusion:**

Furlow primary Z-plasty is a safe and effective operation for symptomatic SMCP with a success rate of 83% with only 5% rate of complications.

Submucous cleft palate (SMCP) is defined as an incomplete union of the mesoderm resulting in the incomplete union of the palatal muscle ring across the soft palate, while the ectoderm fuses, normally resulting in the intact oral and nasal mucosa.^1^ SMCP often presents variably and poses a clinical challenge during postnatal screening, as not all patients present with all 3 features of the traditional Calnan’s triad – a bifid uvula, zona pellucida, and notching in the posterior surface of the hard palate.^2^ Children with SMCP might suffer from a broad spectrum of symptoms, such as feeding difficulties and nasal regurgitation early on, and later speech difficulties caused by velopharyngeal insufficiency (VPI) and repeated episodes of otitis media or hearing loss, resulting from the malfunction of the Eustachian tube.^3,4^ VPI results in hypernasality, nasal air leakage, weakened pressure consonants, and compensatory articulation. It is more obvious after 2 years of age when speaking in full sentences begins.^5,6^ Hypernasality is the most common symptom in VPI, evident in about 50% of patients with SMCP, which usually requires corrective surgical intervention.^4^

Normally, the nasal and oral cavities are separated from one another during speaking in nonnasal sounds and during swallowing by a closing velopharynx.^7^ In SMCP, while the roof of the mouth appears normal, the levator veli palatini muscle is displaced anteriorly onto the notched hard palate, in most cases resulting in inadequate velopharyngeal closure and variable degrees of VPI. While cleft lip and palate are almost always diagnosed in the first year of life, SMCP is less visible and characterized by delays in diagnosis.^8^ In order to avoid developmental and persisting postsurgical compensatory articulation, early detection of primary VPI speech characteristics, prompt initiation of speech therapy, and the decision to operate early enough are essential.^8–11^ Up to 86% of patients with SMCP require palatoplasty because of VPI.^11^

All patients with symptomatic SMCP are offered surgical treatment at the Cleft and Craniofacial Center, Department of Plastic Surgery, Helsinki University Hospital. If children with SMCP have compensatory articulation, they are referred to speech therapy postoperatively. Preoperative speech therapy is not offered by the Cleft and Craniofacial Center; however, because the diagnosis of SMCP is often delayed, speech therapy at patients’ local health centers or schools has often been offered to them before the diagnosis. Furlow double-opposing Z-plasty is our method of choice in the primary repair of SMCP, as well as in secondary speech-correcting surgery. Double-opposing Z-plasty has been efficient in treating VPI as secondary surgery at our cleft center, with less favorable results in syndromic patients.^12,13^ The aim of this single-center retrospective study is to investigate the efficiency and safety of Furlow double-opposing Z-plasty in primary surgery in both nonsyndromic and syndromic children with symptomatic SMCP.

## METHODS

### Participants

This was a retrospective single-center archive study, approved by Helsinki University Hospital (HUH), Finland, following the principles of the Declaration of Helsinki. We searched the HUH patient database for all nonsyndromic and syndromic patients with SMCP who underwent double-opposing Z-plasty with palatal repair as primary surgery between 2008 and 2017.

Four cleft surgeons performed Furlow double-opposing Z-plasty on 48 patients with symptomatic SMCP in our cleft center between 2008 and 2017. We included only those who were operated on by 2 high-volume surgeons, numbering 43. We excluded 3 patients who had primary palatoplasty before undergoing secondary double-opposing Z-plasty for residual VPI. One high-volume cleft surgeon operated on 29 cases and the other on 11. We divided the patients into 2 groups: nonsyndromic and syndromic patients.

Patient characteristics are in Supplemental Digital Content 1, http://links.lww.com/SCS/E985. Of the patients, 22 (55%) were male and 6 (15%) syndromic. Surgeon B operated on significantly more syndromic children (*P*=0.039). Median age at diagnosis was 4.1 (SD 2.0, range 0.1–13.3) years for all, 4.4 (SD 3.0, range 0.4–13.3) years for nonsyndromic, and 3.3 (SD 1.8, range 0.1–4.6) years for syndromic children. Median age at the Furlow Z-plasty was 4.8 (SD 2.6, range 3.1–13.6) years for all, 4.9 (SD 2.7, range 3.1–10.5) years for nonsyndromic, and 4.6 (SD 2.1, range 3.8–9.3) years for syndromic children. The median follow-up time was 19.3 (SD 14.0, range 6.0–57.6) months for all, 18.7 (SD 14.3, range 6.0–57.7) months for nonsyndromic, and 24.9 (SD 12.9, range 6.4–42.1) months for syndromic children. The first postoperative follow-up visit was between 6 and 12 months postoperatively. If velopharyngeal function (VPF) was not competent in the first postoperative speech follow-up visit, a new speech follow-up was organized, usually within 6 to 12 months or within the standard treatment protocol.

Postoperative patient records underwent a search for complications of the Furlow Z-plasty, with recording of all postoperative symptoms. Obstructive sleep apnea (OSA) was noted if the patient had a recorded positive sleep study in the patient records postoperatively.

### Surgical Procedure

**FIGURE 1:**
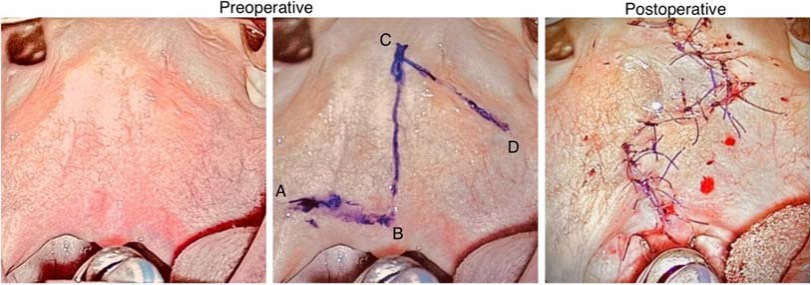
Intraoperative view of Furlow double-opposing Z-plasty.

Preoperative markings for Furlow Z-plasty on the soft palate are presented in Figure 1. The incision for the oral mucosa was based on the original Furlow Z-plasty design, with an anterior limb and posterior limbs.^14^ Finishing incisions were performed after the mobilization of the flap, and tightness of the closure was known. The incision started in the oral layer from point B to C and extended to point A. A full-layer midline incision stretched from the palatal mucosa. On the left side, a myo-mucosal flap was raised from the nasal layer with the levator muscle attached to the oral mucosa. At the level of the levator muscle, no muscle fibers were left attached to the nasal mucosa.

On the right side, incision C to D was performed, and the oral mucosal flap was raised, leaving all muscle fibers attached to the nasal layer. Mirror-image Z-plasty flaps were performed on the nasal layer. On the right side of the soft palate, the levator muscle was rotated into retroposition with the nasal Z-plasty flap. On the left side, a nasal mucosa Z-plasty flap was turned anteriorly. The nasal flaps exchanged places in Z-plasty fashion. Closure of the mucosa was done using 5-0 monocryl sutures. After that, a levator-muscle ring was reconstructed and sutured with 4-0 monocryl or 4-0 PDS sutures. Closure of the oral layer was done in Z-plasty fashion by transposing flap A-B-C anteriorly and B-C-D posteriorly with 4-0 and 5-0 monocryl sutures. For a palatal view after closure, see Figure 1. The surgeons used × 2.5 to ×3.5 magnification loops. Lidocain cum adrenalin was used for hemostasis.

### Speech Assessment

The children were invited to the multidisciplinary cleft team’s follow-up visits at Cleft Palate and Craniofacial Center, Helsinki, as a part of the standard treatment protocol at ages 3, 5, 8, 10, 12, 14, 16, and 18 years. The first postoperative follow-up visit occurred 6 to 12 months after Furlow palatoplasty. A cleft center speech pathologist with years of experience in assessment of cleft speech evaluated the patients’ speech with tests, and spontaneous speech during conversation with the child. Perceptual evaluation of speech involved a Finnish naming test originally designed for the Scandcleft project^15^ containing single words and sentences particularly vulnerable to the cleft condition. A speech pathologist evaluated typical cleft speech characteristics: hypernasality, audible nasal emissions, weakness in pressure consonants, oral stops or fricatives (in Finnish /*P*/, /t/, /k/, /s/), and compensatory articulation (glottal, and nasal or pharyngeal fricatives). Most children aged over 5 years underwent assessment of nasality with nasometry (Kay Pentax Nasometer II, model 6400, Kay Elemetrics, Lincoln Park, NJ, USA, 2001). Nasometry was used during a speech test containing sentences vulnerable to VPI speech characteristics, including pressure consonants and high vowels. In the Finnish language, more than 29% score in nasometry indicates hypernasality.^16^ Nasoendoscopy and videofluoroscopy were methods often needed in planning VPI surgery but not routinely in evaluating VPI.

The speech pathologist divided patients into 4 groups depending on VPF: (0): competent, (1): borderline competent, (2): mild-to-moderate VPI, and (3): severe VPI (Supplemental Digital Content 2, http://links.lww.com/SCS/E985). We retrospectively evaluated preoperative and postoperative speech data from hospital records and then pooled the 4 possible ratings into 3 scale values corresponding to the traffic light color coding system,^17^ where a green color indicates competent or borderline competent VPF with normal/no speech deviances or slight deviances/single occurrences within the normal limit (value 0 and 1), yellow indicates mild VPI with mild deviation/some occurrences (value 2), and red indicates moderate-to-severe VPI with moderate deviation/frequently occurring and severe/occurs always or almost always (value 3) (Supplemental Digital Content 2, http://links.lww.com/SCS/E985). Statistical analyses were performed using the 3-value traffic light color coding scale.

### Speech Therapy

Speech therapy was recorded if the child received speech therapy pre or postoperatively. The type of speech therapy was designed to target speech problems concerning the cleft diagnosis, such as compensatory articulation, phonological delay, difficulties with articulation, and speech characteristics related to VPI, but also other speech deficiencies. Complete documentation of the speech therapy targets and amount were unavailable, as speech therapy may have been provided by local hospitals or schools.

### Statistical Analysis

Data analysis used SPSS version 24 (IBM Corp., Armonk, NY, USA). The χ^2^ and Fisher tests served to compare results between groups. Wilcoxon’s signed-rank test was used to compare VPI classifications pre- and postoperatively within the same group. We considered a probability of less than 0.05 significant.

## RESULTS

### Velopharyngeal Function

**FIGURE 2:**
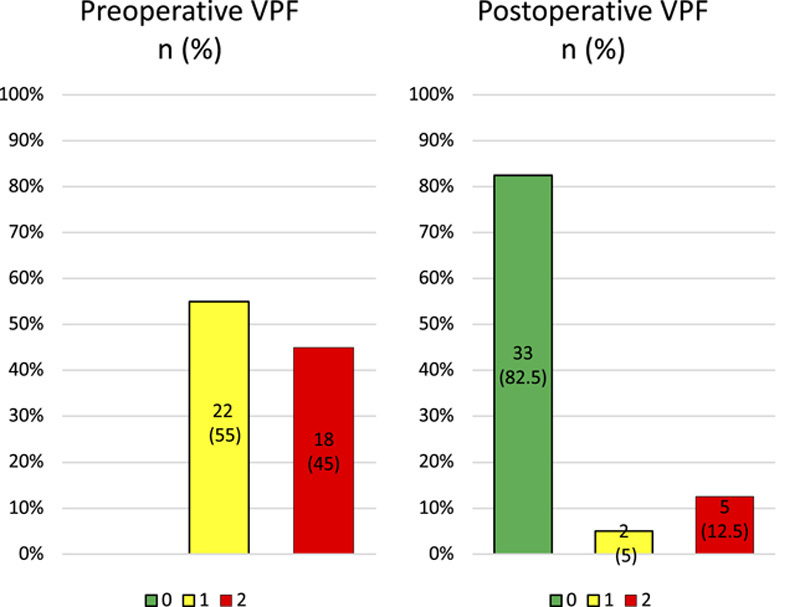
Preoperative and postoperative velopharyngeal function of all 40 children.

The preoperative and postoperative results among all children who underwent Furlow Z-plasty are in Figure 2. Competent or borderline competent VPF (green color) was accomplished in 82.5% of children. A statistically significant improvement resulted in preoperative and postoperative ratings of VPF (*P*<0.00). Postoperative VPF did not differ whether the surgery was performed before or after the age of 5 years (*P*=0.220). Postoperative results of nonsyndromic and syndromic children are in Supplemental Digital Content 3, http://links.lww.com/SCS/E985. Of the patients, 34 of 40 were nonsyndromic, and 29 (85%) of them achieved competent or borderline competent VPF postoperatively. Six of 40 patients had a syndrome, and 4 (67%) of them achieved competent or borderline competent VPF. No significant differences emerged in postoperative VPF between nonsyndromic and syndromic children (*P*=0.279).

### Speech Therapy

Preoperatively, 45% of patients received speech therapy, including 67% of syndromic and 41% of nonsyndromic patients. Postoperatively, 43% of patients received speech therapy, including 84% of syndromic and 35% of nonsyndromic patients.

### Complications

Complications of the Furlow Z-plasty arose in only 2 (5%) patients including a mild wound dehiscence noted at 7 days postoperatively which had completely healed by the postoperative follow-up visit and 1 fistula at the junction of the hard and soft palate noted at 1 year postoperatively. The fistula did not require surgical repair. No patient received an OSA diagnosis postoperatively.

### Secondary Operations

After Furlow Z-plasty, 4 (10%) patients needed additional surgical treatment for VPI: 1 received palatal re-repair with another double-opposing Z-plasty and 3 received a pharyngeal flap. One of the patients required another reoperation for revision of the pharyngeal flap. Two of the 4 patients requiring a secondary operation for VPI were syndromic, but no significant difference (*P*=0.100) was found in reoperation rate between syndromic and nonsyndromic patients (Supplemental Digital Content 3, http://links.lww.com/SCS/E985).

## DISCUSSION

We explored the operative results of 40 children with symptomatic SMCP treated with Furlow double-opposing Z-plasty and found that it was very effective in correcting VPI in 83% of patients, with no significant differences between syndromic and nonsyndromic patients, although the success rate in syndromic children was 67% and in nonsyndromic children 85%. The complication rate was 5%, and only 10% of patients needed secondary speech-correcting surgery.

Surgical intervention for SMCP repair still lacks good clinical comparative studies, and many methods for surgical treatment exist. Since some patients with SMCP develop completely normal speech with velopharyngeal competence, repair is usually done only if speech problems become apparent;^18^ however, there is still an ongoing debate as to whether to operate early or only when speech problems become apparent.^11^ At the Helsinki Cleft and Craniofacial Center, patients with symptomatic SMCP are offered surgical treatment. The aim of SMCP repair should be to restore velopharyngeal competence, which can be done by restoring palatal muscles into a more anatomical dorsal position to achieve better function and velar mobility, possibly combining this with palatal lengthening, or restricting the passage between the oropharynx and nasopharynx.^8,18,19^ Many methods for surgical intervention for symptomatic SMCP have been used: excision of the submucous zone with primary closure, intravelar veloplasty, pharyngeal flap, palatal pushback procedures, palatal lengthening procedures such as Furlow Z-plasty, or combination of 2 methods.^18^ A systematic review of surgical treatment of SMCP based on perceptual and instrumental analysis by Gilleard and colleagues concluded that pharyngeal flap and palate reconstruction (including Furlow and radical muscle retropositioning) are currently the methods most commonly used to treat VPI in children with SMCP, but due to variability in methods of speech outcome reporting and diverse populations, the review was unable to demonstrate the superiority of any 1 surgical method.^20^ In the review by Gilleard et al, the results in achieving normal speech were described as follows: Furlow Z-plasty 67% to 97%, muscle correction/retropositioning 30% to 33%, pharyngeal flap 32% to 100%, and sphincter pharyngoplasty 50% to 72%.^20^ As they concluded, great variability exists in the methods and grading of speech outcome reporting: for example, this review showed normal speech in only 33% after Sommerlad’s muscle correction method, although in Sommerlad’s original study 63% achieved normal resonance or only mild and occasional hypernasality, which is usually seen as a successful operative result.^20,21^

Authors who advocate performing pharyngeal flap primarily believe that such surgery has the highest chance of success in correcting VPI in 1 operation.^22,23^ However, studies have raised concerns about the risk of OSA after a pharyngeal flap.^24–28^ On the other hand, the idea behind palate reconstruction (including intravelar veloplasty and Furlow Z-plasty) in SMCP repair is that these methods retain normal anatomy, which is the first step in retaining velopharyngeal competence. Furthermore, Furlow Z-plasty also achieves palatal lengthening. These methods do not compromise the airway, and most of the children can avoid more obstructive procedures. However, a pharyngeal flap as secondary surgery is still possible if residual VPI exists.

The double-opposing Z-plasty was first introduced by Leonard Furlow in 1986 for primary cleft repair.^14^ Later, Randall introduced the method as a secondary operation^29^ and Chen revealed that it can be used to treat SMCP.^30^ Furlow double-opposing Z-plasty aims to lengthen the palate while preventing longitudinal scar contracture with Z-plasty. In this technique, the levator muscle sling is reconstructed, and the anatomical reorientation of the levator muscle allows better velar movement and function. Previously reported success rates after Furlow Z-plasty in patients with symptomatic SMCP range from 67 to 97%.^19,30–32^ Our result of 82.5% success rate is in line with the previous studies; furthermore, in our study population, only 10% required secondary speech-correcting surgery. There is great variety in previously reported need for secondary speech-correcting surgery after Furlow Z-plasty in patients with SMCP: rates range from 0% to 33%.^9,31,33–38^ The greatest advantage of Furlow Z-plasty is its low risk of associated OSA postoperatively compared to the pharyngeal flap technique. Previous studies comparing pharyngeal flap and Furlow Z-plasty procedures as secondary surgeries reported OSA in 78% to 96% after pharyngeal flap and 25% to 35% after Furlow Z-plasty.^25,39^ In our study, no patient received an OSA diagnosis postoperatively; however, sleep studies were not done routinely because of the retrospective study design.

The timing of double-opposing Z-plasty in the present study showed a median operation age of 4.8 years, which is slightly older than the 4.5 years indicated by Smarius and colleagues.^11^ Sullivan et al recommend double-opposing Z-plasty as the primary operation for children younger than 4 to 5 years with SMCP and VPI.^19^ In our study, the operation age of syndromic patients is slightly lower at 4.5 years and nonsyndromic 4.9 years. The optimal timing of SMCP repair is debated, but the necessity of obtaining an accurate assessment of speech before a therapeutic decision is made cannot be overemphasized. This assessment requires the cooperation of the patient and usually cannot be adequately made until the patient has reached the age of 3. In this study, children with SMCP present late at 4.1 years of age, which appears to be earlier than in the study by Reiter et al,^4^ but later than by Smarius et al^11^ which found the median age of diagnosis to be 4.9 and 3.7 years, respectively. However, the studied population of the present study was limited to children who were surgically treated, therefore the mean age does not represent the whole population of children with SMCP. As the diagnosis of SMCP is often delayed, most children have received speech therapy at their local health centers and schools; as did 45% of the studied children. At the Helsinki Cleft and Craniofacial Center, we offer speech therapy postoperatively for children with symptomatic SMCP only if they are suffering from compensatory articulation, however, some of these children might have other speech problems, such as delayed speech, which might be treated with speech therapy. Postoperatively, 43% of the studied children did receive speech therapy, however, it might have been given because of other speech problems unrelated to the SMCP condition and VPI. Unfortunately, since the retrospective nature of the study and because speech therapy is often given in local health centers and schools, we lack further details of the given speech therapy.

The study also reports syndromic patients at a rate of 12.5%, whereas previously published rates tend to be higher from 18% to 32%.^4,11,19^ Again, our rate includes only surgically treated children. Syndromes might be correlated with the greater need for secondary speech-correcting surgeries^31^ and limited success of speech therapy after Furlow palatoplasty treatment of SMCP.^38^ Brooker et al showed a respectable review of 351 patients with SMCP undergoing Furlow Z-plasty and found a need for secondary speech-correcting surgery to be significantly higher at 24.4% in syndromic versus 9.2% in nonsyndromic patients. In our study, there was no significant difference in speech outcomes among syndromic versus nonsyndromic groups even though our results are similar to those Brookers et al: syndromic children had lower success rate (67% versus 85%) and more reoperations (33% versus 6%) than nonsyndromic children. The number of syndromic children was low (6 children) which might explain the nonsignificant change, and the difference should be confirmed in future studies with more patients. Furthermore, in our previous studies of the efficiency of double-opposing Z-plasty for secondary speech-correcting surgery in patients with isolated cleft palate and unilateral complete cleft lip and palate, we found a similar trend of double-opposing Z-plasty leading to worse outcomes in syndromic patients compared to nonsyndromic patients.^12,13^

With a centralized cleft unit in Finland, we were able to study a moderate number of patients who had very similar treatment and follow-up protocols. Furthermore, all the procedures studied were performed by just 2 experienced cleft surgeons. Our study reviews surgical outcomes and does not discuss conservatively treated patients. A very experienced speech pathologist assessed the speech but speech evaluations were gathered from the patient’s records, and consequently such a retrospective study design presents a certain methodological weakness. We, unfortunately lack a validated scale of velopharyngeal competence and acknowledge the problem of not using one in studies regarding cleft palate speech assessments.

## CONCLUSIONS

Furlow double-opposing Z-plasty is safe and effective in treating VPI in SMCP patients as a primary surgery, with a success rate of 83% and no severe complications. Its efficiency might be worse in syndromic children, with a success rate of 67% compared with 85% in nonsyndromic children; however, the difference was not statistically significant, and this issue requires further studies with larger patient samples.

## Supplementary Material
