# Supplementary material for: Outcomes of Primary Furlow Double-Opposing Z-plasty for the Treatment of Symptomatic Submucous Cleft Palate
Source: J Craniofac Surg. 2023 May 24;34(7):2066–70. doi: 10.1097/SCS.0000000000009385 (PMC10521778; doi:10.1097/SCS.0000000000009385)
Supplement: Supplementary file 1 [file scs-34-2066-s001.docx]

**Supplemental digital content**

Supplemental digital content 1. Characteristics of all, nonsyndromic, and syndromic patients.

|  | All 40 (%) | Nonsyndromic 34 (%) | Syndromic 6 (%) | P |
| --- | --- | --- | --- | --- |
| Syndrome  Catch-22  OFCD  CCHS  ODDD  47 XXX Syndrome  6q Duplication Syndrome | 0 (0)  1  1  1  1  1  1 |  | 6 (100)  1  1  1  1  1  1 | 1.000 |
| Gender  Male  Female | 22 (55)  18 (45) | 19 (56)  15 (44) | 3 (50)  3 (50) | 1.000 |
| Age*  <5  >5 | 23 (57.5)  17 (42.5) | 18 (53)  16 (47) | 5 (83)  1 (17) | 0.165 |
| Surgeon  A  B | 29 (72.5)  11 (27.5) | 27 (79)  7 (21) | 2 (33)  4 (66) | 0.039 |
| Preoperative VPF  0 Green  1 Yellow  2 Red | 0  22 (55)  18 (45) | 0  20 (59)  14 (41) | 0  2 (33)  4 (66) | 0.381 |

^*^Age at Furlow Z-plasty

OFCD, Oculofaciocardiodental syndrome

CCHS, Congenital central hypoventilation syndrome

ODDD, Oculodentodigital Syndrome

VPF, Velopharyngeal function

P, Difference between nonsyndromic and syndromic patients

Supplemental digital content 2. Overall velopharyngeal function (VPF) graded by speech pathologist.

| 0 | Competent | Clear speech without any hypernasality, nasal emissions, or difficulties producing pressure consonants. |
| --- | --- | --- |
| 1 | Borderline competent | Mild and temporary hypernasality or nasal emissions with accurate pressure consonants. |
| 2 | Mild-to-moderate VPI | Constant mild-to-moderate hypernasality, with possible nasal emissions, but accurate pressure consonants. |
| 3 | Moderate-to-severe VPI | Consistent moderate-to-severe hypernasality with possible nasal emissions. Pressure consonants were either weak or replaced by glottal stops. |

Color refers to the traffic light color coding system used in the results. Green refers to competent or borderline competent VPF, yellow to mild VPI, and red to moderate-to-severe VPI.

Supplemental digital content 3. Postoperative velopharyngeal function (VPF) and re-operations of nonsyndromic and syndromic children.

|  | Nonsyndromic  n=34 (%) | Syndromic  n=6 (%) | p |
| --- | --- | --- | --- |
| Postop VPF |  |  | 0.279 |
| 0 | 29 (85) | 4 (67) |  |
| 1 | 2 (6) | 0 |  |
| 2 | 3 (9) | 2 (33) |  |
| Re-operations | 2 (6) | 2 (33) | 0.100 |
| Re-repair | 0 | 1 |  |
| Pharyngeal flap | 2 | 1 |  |

Green refers to competent or borderline competent VPF, yellow to mild VPI, and red to moderate-to-severe VPI.
